# Identification of markers for migrasome detection

**DOI:** 10.1038/s41421-019-0093-y

**Published:** 2019-05-21

**Authors:** Xiaoxin Zhao, Yaxin Lei, Jiajia Zheng, Junya Peng, Ying Li, Li Yu, Yang Chen

**Affiliations:** 1 The State Key Laboratory of Membrane Biology, Tsinghua University-Peking University Joint Center for Life Sciences, School of Life Sciences, Tsinghua University, Beijing, 100084 China; 2 Department of Laboratory Medicine, Peking University Third Hospital, Beijing, 100191 China; 3 Medical Research Center, Peking Union Medical College Hospital, Beijing, 100043 China; 4 Tsinghua University-Peking University Joint Center for Life Sciences, technology center for protein sciences, School of Life Sciences, Tsinghua University, Beijing, 100084 China; 5 Center for Precision Medicine Multi-Omics Research, Peking University Health Science Center, Peking University, Beijing, 100191 China

**Keywords:** Organelles, Proteomic analysis

Dear Editor,

The migrasome is a newly discovered cellular organelle in migrating cells, first described in 2015^1,2^. Migrasomes have been proposed to mediate cell-cell communications and may exert physiological and pathological effects. In zebrafish embryos, generation of migrasomes has been observed during gastrulation, and migrasomes have been shown to be essential for organ morphogenesis during embryonic development (Personal communication). Although migrasome-like structures have been detected by transmission electron microscopy (TEM) in various organs including eye, lung and intestine, definitive evidence for the presence of migrasomes in vivo in mammals is still lacking. Exosomes are defined as 30–100 nm extracellular vesicles derived from the multivesicular body and are secreted by a variety of cell types. Exosomes have attracted great attention in clinical studies since they were detected in solid tissues, such as cartilage^3^, and in biological fluids, such as blood^4^. One of the most commonly used methods for detection of extracellular vesicles (EVs) is a biochemical approach using known marker proteins. This strategy allows researchers to identify EVs in complex biological samples such as body fluids and tissues, and thus provides great flexibility for detection of EVs in vivo. So far, there is no satisfactory marker for biochemical detection of migrasomes. TSPAN4 and integrin are known to be enriched on migrasomes^1,5^. However, both proteins are also present on exosomes^6^, making it difficult to distinguish the two structures biochemically based only on detection of these proteins. Imaging is the only approach for confirming the presence of migrasomes, but it is not compatible with most clinical samples. Thus, there is an urgent need to identify migrasome-specific markers.

Our strategy to identify migrasome-specific protein markers is to select candidate proteins based on quantitative mass spectrometry analysis on purified migrasomes and exosomes, followed by verification using cell biology and biochemistry approaches. To generate migrasomes, we used NRK cells stably expressing TSPAN4-GFP (Supplementary Fig. S1a). The crude migrasome extract was resuspended in 19% density medium and subjected to density gradient centrifugation. Migrasomes floated to an interval between 10–15% (Supplementary Fig. S1b, fractions 4–6). Western blotting analysis confirmed the presence of the migrasome markers TSPAN4 (labeled by GFP) and integrin α5 in fractions 4–6 (Supplementary Fig. S1c). Exosomes were purified using conventional approaches and verified by TEM and western blotting analysis^7^ (Supplementary Fig. S1d–f). The structures of migrasomes and exosomes were compared by TEM and cryo-EM analyses (Fig. 1a, b). Migrasomes are phospholipid bilayer organelles with diameters of~500 nm and some purified migrasomes were still attached to retraction fibers. Exosomes are ~100 nm in diameter. Thus, the two structures are different in morphology and size (Supplementary Fig. S1g).

**Fig. 1:**
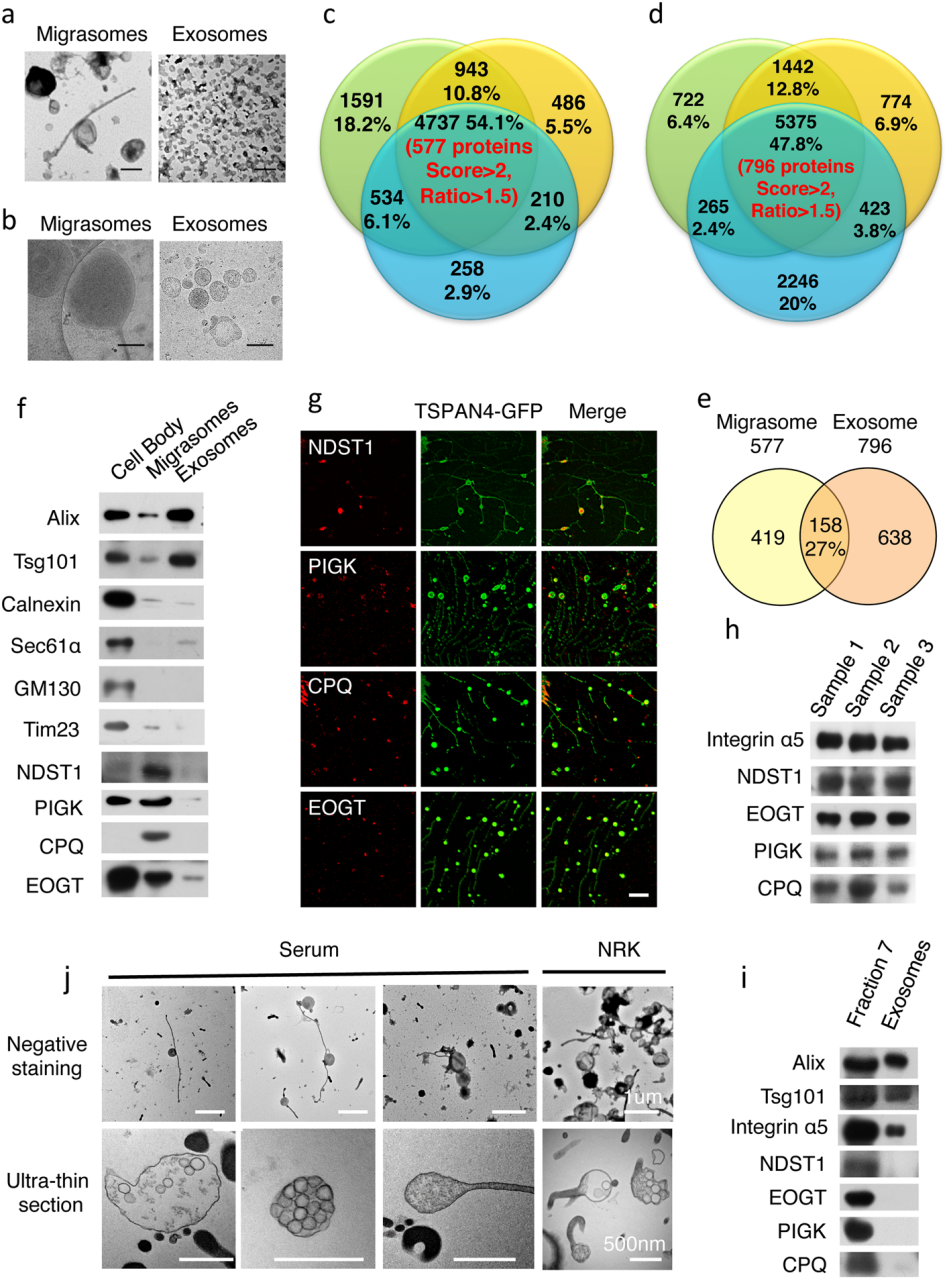
Identification of migrasome-specific markers based on quantitative mass spectrometry. **a** Representative TEM images of negatively stained samples of migrasomes and exosomes purified from NRK cells overexpressing TSPAN4-GFP. Scale bar, 500 nm. **b** Representative cryo-EM images of migrasomes and exosomes purified from NRK cells overexpressing TSPAN4-GFP. Scale bar, 200 nm. **c** Overlap of proteins from migrasomes identified in three independent quantitative mass spectrum analyses. The central overlapping area shows the 4737 proteins that were reproducibly detected in all three analyses. 577 of these proteins have score >2 and ratio >1.5. **d** Overlap of proteins from exosomes identified in three independent quantitative mass spectrum analyses. The central overlapping area shows the 5375 proteins that were reproducibly detected in all three analyses.796 of these proteins have score >2 and ratio >1.5. **e** Venn diagram showing the number of proteins enriched on migrasomes and exosomes with score >2 and ratio >1.5 in all three repeats. The total number of proteins is shown above the diagram, and the unique proteins and shared proteins are shown in the circles. **f** Samples from cell bodies and purified migrasomes and exosomes were analyzed by western blotting using antibodies against Alix, Tsg101, Calnexin, Sec61α, GM130 and Tim23, and identified migrasome-specific marker NDST1, PIGK, CPQ and EOGT. **g** NRK cells stably expressing TSPAN4-GFP were cultured on a fibronectin-coated surface. NDST1, PIGK, CPQ and EOGT were detected by immuno-fluorescence using the corresponding antibodies. Scale bar: 5 μm. **h** Serum samples from three different individuals (1 ml/Sample) were subjected to centrifugation at 20000 × *g*. The pellet was analyzed by western blot using antibodies against indicated proteins. **i** Fraction 7 from Supplementary Fig. 4B and exosomes purified from serum samples were analyzed by antibodies against Alix, Tsg101, Integrin β1, NDST1, EOGT, PIGK and CPQ. **j** Representative TEM images of negatively stained samples and embedded ultra-thin sections of migrasomes purified from human serum and from NRK cells stably expressing TSPAN4-GFP. Scale bars are indicated in the figure

We employed tandem mass tag (TMT) labeling followed by quantitative mass spectrometry to identify proteins enriched on migrasomes. Purified migrasomes and cell bodies were labeled with different tags of different mass. The samples were then combined and analyzed to determine the proteins enrichment on migrasomes compared to cell bodies (Supplementary Fig. S2a). Three independent migrasome preparations were analyzed by TMT-based quantitative mass spectrometry. Of the total protein hits, 4737 (54.1%) were reproducibly detected. Of these, we selected 577 proteins which met the criteria that protein score in SEQUEST software >2 and migrasome-to-cell-body ratio > 1.5 in each of the three data sets (Fig. 1c). The enrichment of the proteins on migrasomes is 1.5 times compared to cell bodies. GO classification showed that the genes encoding these 577 proteins are enriched for GO terms includingcell migration, cell-substrate adhesion, lipid catabolicprocesses as well as protein glycosylation and glycoprotein metabolic processes (Supplementary Fig. S2b). We acquired exosome proteomic data using the same approach and selected a list of 796 proteins enriched on exosomes using the same criteria (Fig. 1d). The comparison of migrasome and exosome proteomics showed that the two structures shared only 158 (27%) proteins (Fig. 1e).

Base on the MS data, we screened for proteins enriched only on migrasomes using purified migrasomes and exosomes samples. We first confirmed that purified migrasomes and exosomes contain known protein markers without contamination of other cellular organelles (Fig. 1f). Western blotting results showed that four proteins, NDST1 (bifunctionalheparan sulfate N-deacetylase/N-sulfotransferase 1), PIGK (phosphatidylinositol glycan anchor biosynthesis, class K), CPQ (carboxypeptidase Q) and EOGT (EGF domain-specific O-linked N-acetylglucosaminetransferase), were enriched in migrasomes, but were absent or barely detectable in exosomes (Fig. 1f, Supplementary Fig. S3a). We observed that mcherry-labeled four proteins localized on migrasomes with live cell imaging (Supplementary Fig. S3b). We also stained cells with specific antibodies against these proteins, and found that all of them are indeed localized in migrasomes (Fig. 1g).

With these markers available, we next set out to detect migrasomes in human serum. We first took 1 ml serum samples each from three individuals and found that all four proteins and integrin α5 were detected (Fig. 1h), indicating that migrasomes may be present in serum. We further fractionated human serum using a protocol similar to that for isolating migrasomes from cultured cells (Supplementary Fig. S4a), and found that fraction 7 contained all 4 identified migrasome markers and integrin α5 (Supplementary Fig. S4b), which strongly suggests that migrasomes are present in serum. Next, we compared fraction 7 and purified exosome fraction from serum samples and found that all four migrasome markers were exclusively present in fraction 7 but not in exosomes, which is consistent with the marker specificity in detecting migrasome obtained from cultured cells (Fig. 1i).

To directly verify the presence of migrasomes in serum, we subjected the sample from fraction 7 to negative staining and TEM revealed vesicles attached to tubules, which are characteristic morphological features of migrasomes (Fig. 1j). The diameter of the structures purified from serum was very similar to migrasomes purified from NRK cells (Supplementary Fig. S4c). We also embedded the sample from fraction 7 and subjected it to ultra-thin section TEM. This uncovered another characteristic morphological feature of migrasomes, which is the presence of small luminal vesicles within larger vesicles (Fig. 1j). From these data, we conclude that migrasomes are present in human serum.

In this manuscript, we compared the protein composition of migrasomes and exosomes by quantitative MS analysis. Using this information, we successfully identified four marker proteins for migrasomes, which are not present in exosomes. Identification of these migrasome-specific markers enabled us to detect the presence of migrasomes biochemically. Our study identified a set of markers for biochemical detection of migrasomes that is compatible with clinical samples, and will potentially be important in future disease-related studies. Moreover, it may open avenues for translational studies of migrasomes in diagnostic, prognostic and therapeutic applications.

Using this approach, we surprisingly found that migrasomes are present in human serum. The presence of migrasomes immediately raises several questions. Where are migrasomes generated, and by which cell type? How do migrasomes enter the circulatory system? Where do migrasomes go and what is their function in serum? Are serum migrasomes related to certain diseases, and can they be used as a diagnostic tool? Future investigations are needed to address these questions.

## Supplementary information

Supplementary information

## Supplementary information

**Supplementary Information** accompanies the paper at (10.1038/s41421-019-0093-y).
