# Author Correction: Identification of markers for migrasome detection

**DOI:** 10.1038/s41421-022-00404-3

**Published:** 2022-04-06

**Authors:** Xiaoxin Zhao, Yaxin Lei, Jiajia Zheng, Junya Peng, Ying Li, Li Yu, Yang Chen

**Affiliations:** 1 The State Key Laboratory of Membrane Biology, Tsinghua University-Peking University Joint Center for Life Sciences, School of Life Sciences, Tsinghua University, Beijing, 100084 China; 2 Department of Laboratory Medicine, Peking University Third Hospital, Beijing, 100191 China; 3 Medical Research Center, Peking Union Medical College Hospital, Beijing, 100043 China; 4 Tsinghua University-Peking University Joint Center for Life Sciences, technology center for protein sciences, School of Life Sciences, Tsinghua University, Beijing, 100084 China; 5 Center for Precision Medicine Multi-Omics Research, Peking University Health Science Center, Peking University, Beijing, 100191 China

**Keywords:** Organelles, Proteomic analysis

Correction to: *Cell Discovery* (2019) 5:27

10.1038/s41421-019-0093-y Published online 21 May 2019

In the original publication of this correspondence^1^, the dataset identifier in “Sample preparation and quantitative mass spectrometry analysis” section in the “Material and Method” was wrong. The corrected dataset identifier appears below:

The proteomics data were deposited into the ProteomeXchange Consortium via the PRIDE partner repository with the dataset identifier PXD026960.
