# Supplementary material for: Identification of markers for migrasome detection
Source: Cell Discov. 2019 May 21;5:27. doi: 10.1038/s41421-019-0093-y (PMC6527679; doi:10.1038/s41421-019-0093-y)
Supplement: Supplementary file 1 — Supplementary information [file 41421_2019_93_MOESM1_ESM.pdf]

Supplementary Fig. S1

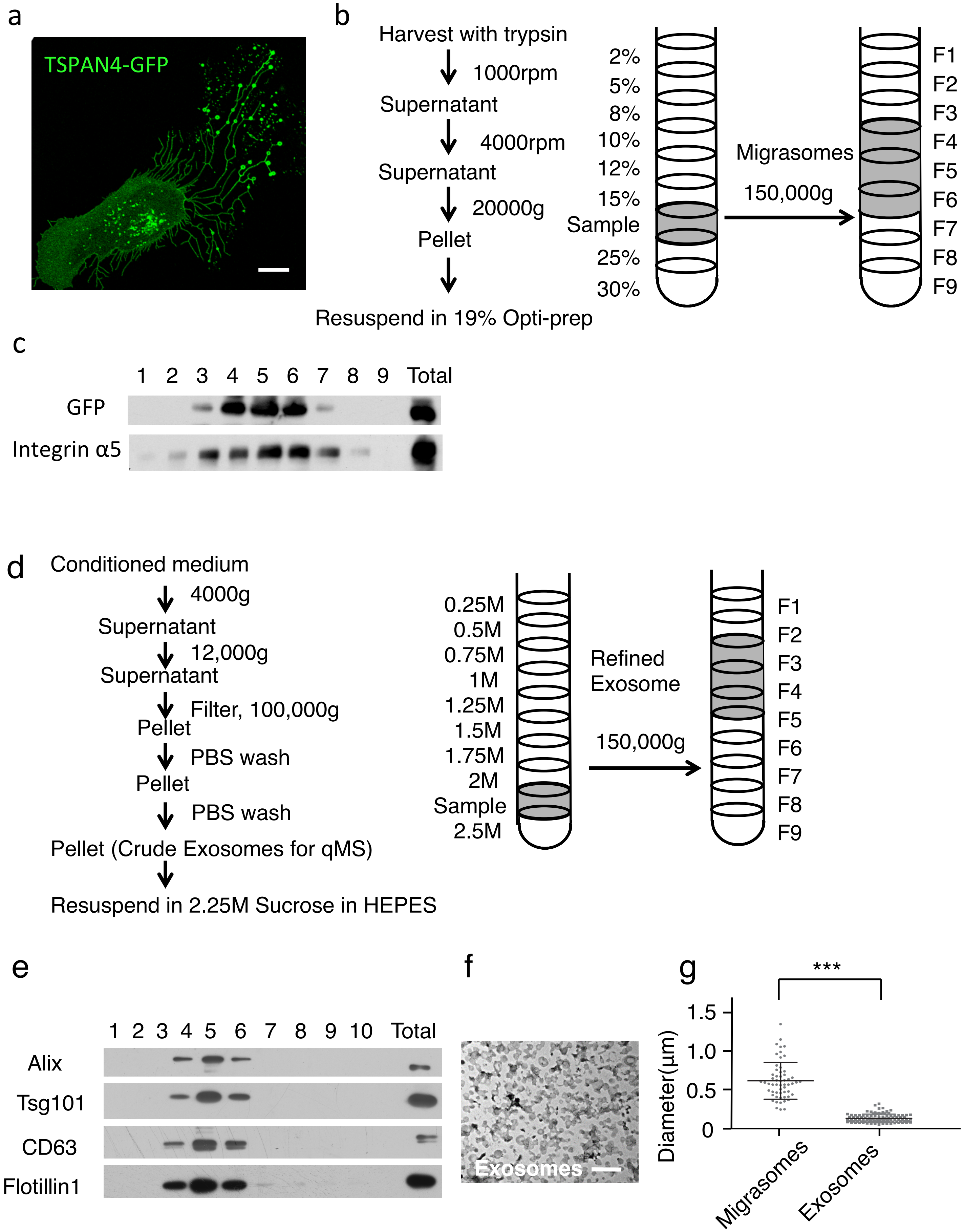

Supplementary Fig. S1. Purification of migrasomes and exosomes.

a NRK cells stably expressing TSPAN4-GFP were cultured on plates coated with fibronectin and then observed by confocal microscopy. Scale bar, 5 µm. **b** Schematic illustration of the refined migrasome purification procedure from tissue cultured cells. **c** Density gradient fractions from b were analyzed by western blotting using an antibody against GFP to detect TSPAN4-GFP and integrin α5. “Total” indicated sample before density gradient marked in b. **d** Schematic illustration of the density gradient centrifugation procedure which was adapted for purification of exosomes from tissue cultured cells. **e** Density gradient fractions from d were analyzed by western blotting using antibodies against exosome specific markers Alix, Tsg101, CD63 and Flot1. “Total” indicated sample before density gradient marked in d. **f** Representative TEM images of negatively stained samples of exosomes. Scale bar, 200nm. **g** Quantification of the average diameter of purified migrasomes and exosomes based on TEM images of negatively stained samples. Each dot represents an individual vesicle. Error bars represented average $\pm$ standard deviation. $P<0.001$.

Supplementary Fig. S2

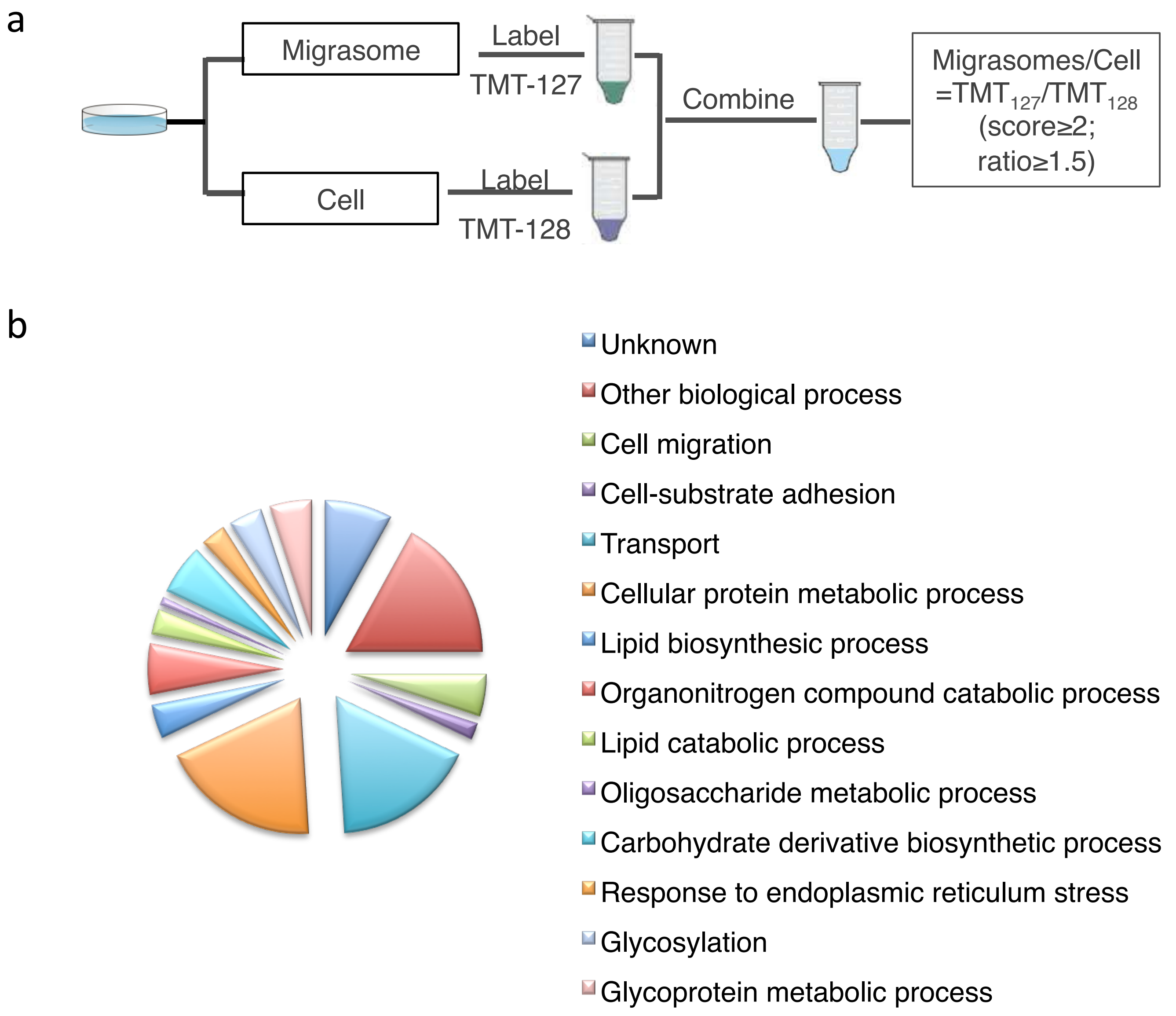

Supplementary Fig. S2. Quantitative mass spectrometry analysis of migrasomes and exosomes

a Schematic illustration of TMT labeling and quantitative mass spectrometry of purified migrasomes and cell bodies. **b** Biological process analysis of the 577 proteins with protein score in SEQUEST software>2 and migrasome-to-cell-body ratio>1.5.

Supplementary Fig. S3

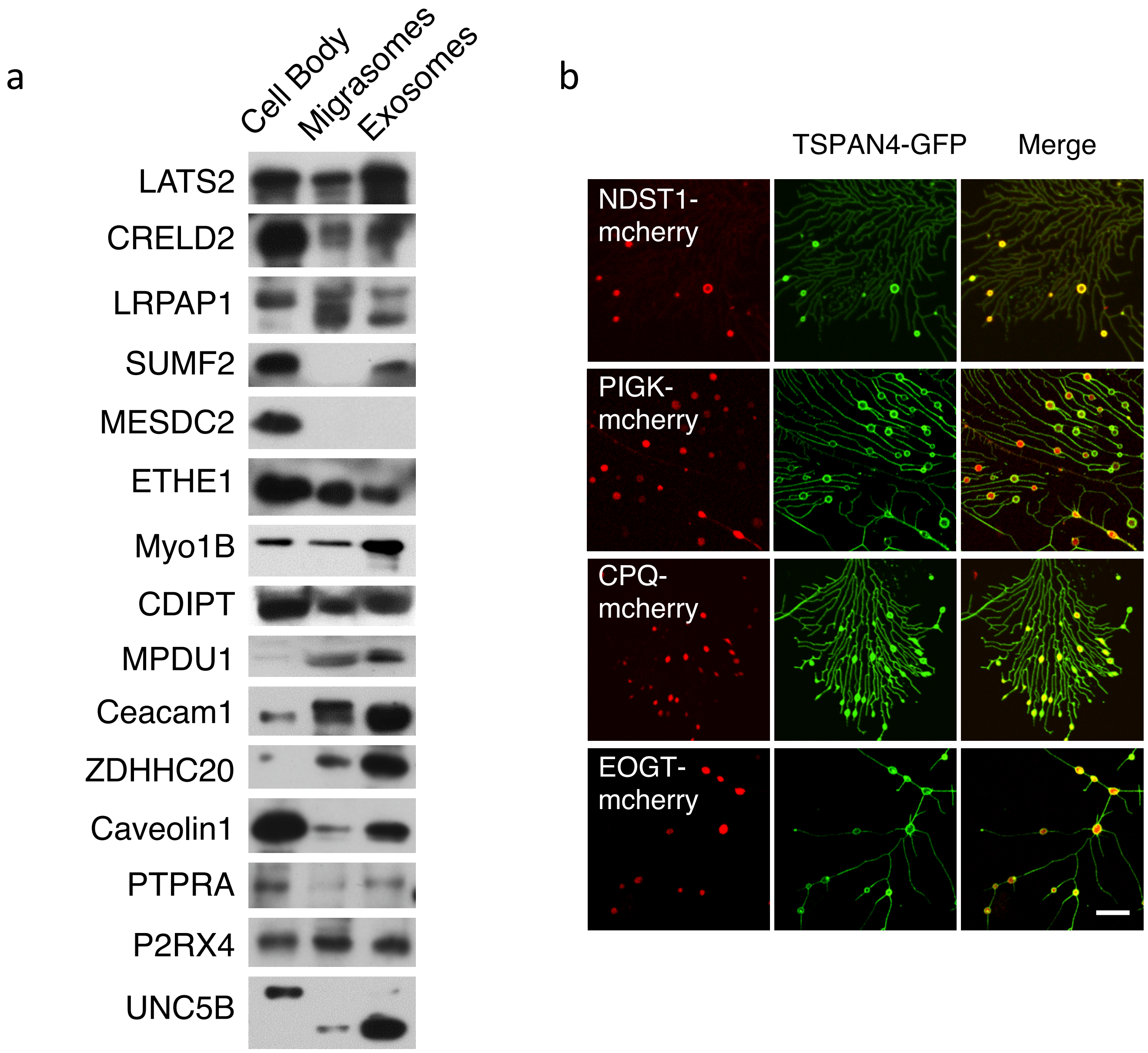

Supplementary Fig. S3. Screening and verifying of migrasome specific markers candidate derived from quantitative mass spectrum
a Samples from cell bodies, purified migrasomes and purified exosomes from NRK cells stably expressing TSPAN4-GFP were analyzed by western blotting using the indicated antibodies. **b** L929 cells overexpressing TSPAN4-GFP were transfected with NDST1-mcherry, PIGK-mcherry, CPQ-mcherry and EOGT-mcherry respectively. Cells were cultured on fibronectin-coated surface for live cell imaging. Scale bar: 5μm.

Supplementary Fig. S4

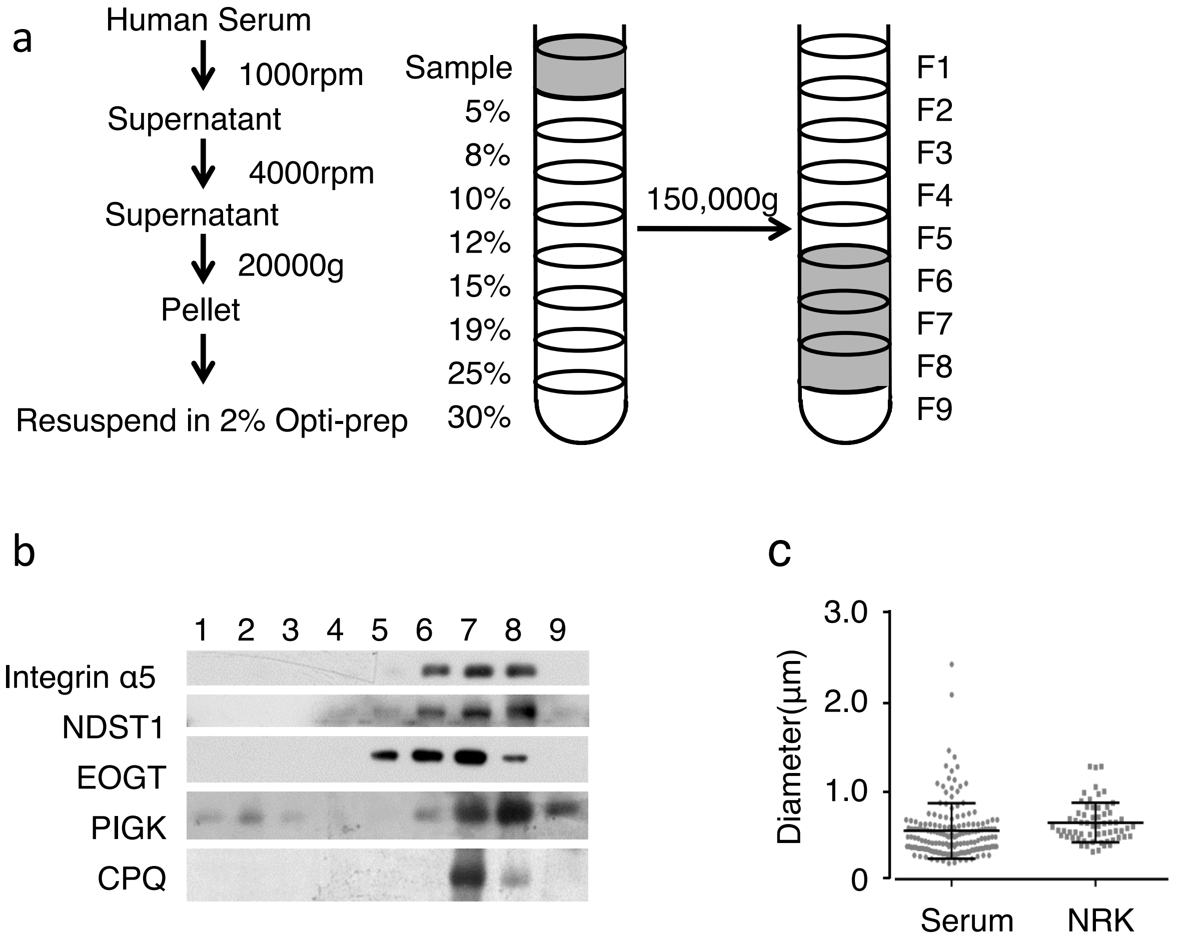

Supplementary Fig. S4. Detection of migrasomes in serum
a Schematic illustration of fractionation procedures for human serum samples. Blood samples for research purposes werecollected in pro-coagulation vacuum tubes using standard venepuncture protocols. Human serum was withdrawn from the tube for the subsequent procedures. **b** Density gradient fractions from a were analyzed by western blotting using antibodies against integrin α5, NDST1, PIGK, CPQ and EOGT. **c** Quantification of the average diameter of purified migrasomes from serum samples and purified migrasomes from NRK cells based on TEM images of negatively stained samples. Each dot represents an individual vesicle. Error bars represented average ± standard deviation. $P>0.05$

Materials and Methods:

Cell lines and antibodies

NRK cells stably expressing Tspan4-GFP were cultured in DMEM with 10% FBS (BI) (5% CO_2). For migrasome production, the culture dishes were coated with fibronectin (0.1-1 μg/ml) (PHE0023, Gibco) for 0.5-2 hours before cells were added . Cells were seeded to 40%confluency in 150mm dishes containing 20ml of growth medium and grown to 60% (~12 hr). For exosome production, cells were seeded to 50%confluency in 150mm dishes (NEST)containing 20ml of growth medium and grown to 100% confluency(~18 hr). Primary antibodies were: anti-ALIX, ab186429 (Abcam); anti-Tsg101, GTX70255 (GeneTex); anti-flotillin1, 610820 (BD); anti-integrin α5, #4705 (Cell Signaling); anti-SDCBP, S1701-25UL (Sigma); anti-β-actin, 200068-8F10-25ul (Zen BioScience); Anti-EXOG, HPA013609-100UL (Sigma); Anti-CPQ, HPA023235-100UL (Sigma); Anti-NENF, ab74474 (Abcam); Anti-PIGK, ab201693 (Abcam); anti-NDST1, SAB1307040-400UL (Sigma); Calnexin (MBL, PM060); Sec61A (CST,14868S); GM130 (BD, 610823).

Serum sample preparation

The participants were recruited from the Medical Examination Center of Peking University Third Hospital in 2018. Blood samples for research purposes were collected in pro-coagulation vacuum tubes using standard venepuncture protocols. Serum was extracted by centrifugation for 10 min at 3000rpm and subsequently stored at −80 °C before use.

Live-cell imaging

Cells were cultured in 35 mm glass-bottom dishes coated with fibronectin (1 μg/ml). Images were acquired using Olympus FV-1000 confocal microscopes.

Immunostaining

Cells grown in 35 mm glass-bottom dishes were fixed with 4% PFA for 10 min at room temperature. Cells were washed with PBS 3 times and permeabilized for 10 min at room

temperature in 0.05% saponin + 10% goat serum in PBS (saponin/FBS). The samples were then blocked with 10% FBS for 10 min at room temperature and incubated with the indicated primary and secondary antibodies at room temperature in 10% FBS.After 3 washes with PBS, the stained samples were directly observed in the 3.5 cm dish using confocal microscopy.

Isolation of migrasomes from tissue culture cells

The isolation procedure was based on a modification of previous protocols (Ma et al, 2014). Briefly, cells were grown in 150mm dishescoated with 0.1 µg/ml fibronectin in full DMEM medium for 12 hours. A batch of purification requires 30-80dishes. The cells and migrasomes on plateswere digested with trypsin and collected in 50ml tubes. All subsequent manipulations were performed at 4°C. Cells and large debris were removed by centrifugation at 1000 g for 10 min followed by 4000g for 20 min. Crude migrasomes were then collected as the pellet by centrifugation at 20,000 g for 30 min. The crude migrasome pellets were washed with PBS and centrifuged down again at 20,000 g for 30min. Migrasome fractionation was performed by density gradient centrifugation, using Optiprep as the density medium (Sigma-Aldrich, D1556). First, a step gradient was built starting with 30%(500 µl), followed by 25%(500 µl), crude migrasomes (19%, 500 µl), 15%(500 µl), 12%(500 µl), 10%(500 µl), 8%(500 µl), 5%(500 µl) and 2%(500 µl). The crude migrasome sample was prepared by first resuspending the pellet with 137.5 µl dilution buffer and then mixing with 400 µl 1 x extraction buffer and 252.5 µl 60% Optiprep. Second, the prepared gradient was centrifuged at 150 000× g for 4 hr at 4°C in an MLS-50 rotor (Beckman). Third, samples were collected from top to bottom (480 µl per fraction). Each fraction was mixed with the same volume of PBS (480 µl) and centrifuged at 20,000g for 30min to collect the pellet. The pellets were washed with PBS and centrifuged again at 20,000g for 30min. The samples were compatible with western blot analysis, TEM, cryo-EM and mass spectrometry.

Isolation of migrasomes from serum samples

Blood samples of normal donors for research purposes werecollected in 6-ml pro-coagulation vacuum tubes using standard venepuncture protocols. Human serum was

withdrawn from the tube for the purification procedures. A total of 50ml of cell-free serum was required for one batch purification. Large debris was removed by centrifugation at 1000 g for 10 min followed by 4000g for 20 min. Crude migrasomes were collected by centrifugation at 20,000 g for 30 min, then washed with PBS. Migrasome fractionation was performed by Iodixanol-sucrose density gradient centrifugation, using Optiprep (Sigma-Aldrich, D1556) as the density medium. First, a step gradient was built starting from 30%(500 µl), followed by 25%(500 µl), 19% (500 µl), 15%(500 µl), 12%(500 µl), 10%(500 µl), 8%(500 µl), and 5%(500 µl). The sample (500 µl) was prepared in 2%Optiprep and layered on top. The gradient was centrifuged at 150 000× g for 4 hr at 4°C in an MLS-50 rotor(Beckman). Samples were collected from top to bottom (480 µl per fraction).Eachfraction was mixed with the same volume of PBS (480 µl), thencentrifuged at 20,000g for 30min. The migrasome pellets were washed with PBS and centrifuged again at 20,000g for 30min. The samples were compatible with western blot analysis, TEM, cryo-EM and mass spectrometry.

Isolation of exosomes from tissue culture cells

The isolation procedure was based on a modification of previous protocols. Briefly, NRK cells stably expressing TSPAN4-GFP were grown on 150mm dishes (NEST) in DMEM media with 10% FBS depleted of exosomes until they reached a confluency of 90-100%. One batch of purification required 20-60 dishes. Conditioned medium was harvested from the cultured cells. All subsequent manipulations were performed at 4°C. Cells and large debris were removed by centrifugation at 4000 g for 20 min followed by 18,000g for 20 min in 50 ml tubes. The supernatant was passed through a 0.2 µm filter and then centrifuged at 100,000 g for 70 min at 4°C. The crude exosome pellet was washed with 60ml PBS, followed by a second step of ultracentrifugation at 100,000 g for 70 min at 4°C to collect the crude exosomes in the pellet.

Sucrose density gradient centrifugation was performed to further purify exosomes. Briefly, crude exosomes were resuspended in 500 µl of HEPES/sucrose stock solution (2.25 M sucrose, 20 mM HEPES/NaOH solution, pH 7.4). The suspension was overlaid with a step sucrose gradient (2.5, 2.0, 1.75, 1.5, 1.25, 1.0, 0.75, 0.5, 0.25 M sucrose, 20 mM HEPES/NaOH, pH 7.4, 500 µl). Crude exosomes (2.25M sucrose, 20mM

HEPES/NaOH) was loaded in between gradient 2M-2.5M sucrose. The gradient was spun at 210,000g at 4°C for 4 hr. Gradient fractions of 500 µl were collected from top to bottom and the exosome pellets were washed in PBS then subjected to a final ultracentrifugation step at 100,000 g at 4°C for 70 min.

Negative staining

Purified migrasome or exosome pellets were resuspended in 50-100 µl PBS, then a 5 µl sample of each was mixed with the same volume of 2.5%glutaraldehyde (PB, pH 7.4), and fixed for 30 min at room temperature. The sample was spread onto glow discharged Formvar-coated copper mesh grids (Electron Microscopy Sciences, Hatfield) for about 5 min, then washed with water. The sample was then stained with uranyl acetate for 2 min. Excess staining solution was blotted off with filter paper and copper mesh grids were washed with water. Post drying, grids were imaged at 10-100 kV using a transmission electron microscope H-7650.

Cryo-electron microscopy

An aliquot of 4 µl of purified migrasomes or exosomes was applied to a glow-discharged QuantifoilR2/2 Cu 300 mesh grid (Quantifoil Micro Tools GmbH, Jena, Germany) blotted inside Vitrobot (FEI, Hillsboro, OR, USA) at 10 °C and plunged into liquid ethane. The specimens were examined in an FEI Tecnai Arctica cryo-electron microscope operated at 200 kV using magnification of 39,000 X or 78,000 X. Images were recorded under low-dose conditions with a dose of ~25 e-/A2 on an FEI Facol II direct electron detector.

Sample preparation and quantitative mass spectrometry analysis

For the quantitative proteomics analysis, samples were performed as described in a previous study. Briefly, proteins of cell bodies, purified migrasomes and purified exosomes were extracted in lysis buffer containing 8 M urea and protease inhibitor (BioTools, Jupiter, Florida) in PBS. Equal amounts of proteins were reduced, alkylated and digested by trypsin, then desalted and labeled with TMT reagents (Thermo, Pierce Biotechnology). The TMT-labeled peptides were mixed and desalted using Sep-Pak C18

cartridges. The tryptic peptides were separated into 12 fractions with high-pH RPLC (XBridgeTM BEH300 C18 5μm, 300Å, 250 mm × 4.6 mm i.d., Waters; mobile phase A (2% acetonitrile, pH = 10.0) and B (98% acetonitrile, pH = 10.0)).

For LC-MS/MS analysis, the peptides were separated by a C18 column (75 μm inner-diameter, 150 mm length, 5 μm, 300 Å) with a Thermo-Dionex Ultimate 3000 HPLC system, which was directly connected with a Thermo Scientific Orbitrap Fusion Lumos Tribrid mass spectrometer. A series of adjusted linear gradients according to the hydrophobicity of the fractions was applied with a flow rate of 300 nL/min. The mass spectrometer was programmed to acquire in the data-dependent acquisition mode. The survey scan was from m/z 375 to 1550 with resolution of 120,000 at m/z 400. After one microscan, the top N most intense peaks with charge state 2 and above were dissociated by normalized collision energy of 35%. The isolation window was set at 0.7 Da width and the dynamic exclusion time was 60 s. The MS2 spectra were acquired with a resolution of 17,500, AGC target of 1e5 and maximum injection time (IT) of 60 ms.

The generated MS/MS spectra were searched against the Uniprot Rat database (January 10, 2015; 89105 sequences) using the SEQUEST searching engine in Proteome Discoverer 1.4 software. The search criteria were as follows: full tryptic specificity was required, one missed cleavage was allowed, carbamidomethylation on cysteine and TMT sixplex on lysine/peptide N-terminals were set as the fixed modifications, oxidation on methionine was set as the variable modification, precursor ion mass tolerances were set at 10 ppm for all MS acquired in the Orbitrap mass analyzer, and the fragment ion mass tolerance was set to 20 mmu for all MS2 spectra acquired. Peptide spectral matches (PSM) were validated using the Percolator provided by Proteome Discoverer software based on q-values at a 1% false discovery rate (FDR). Proteomic analysis was carried out in biological triplicates. Proteins were considered to be differentially expressed with the ratio >0.75 or <1.3 while the p value was lower than 0.05. The proteomics data were deposited into the ProteomeXchange Consortium via the PRIDE partner repository with the dataset identifier PXD020522.

Statistics

Statistical analysis was performed in GraphPad Prism. All data were obtained from at least 3 independent experiments. Error bars in the figures represent the standard deviation (SD). Experimental groups were compared using two-tailed t-tests.
